# Management of Periprosthetic Joint Infection and Extensor Mechanism Disruption With Modular Knee Fusion: Clinical and Biomechanical Outcomes

**DOI:** 10.1016/j.artd.2020.12.008

**Published:** 2021-02-26

**Authors:** Wesley H. Mayes, Anna C. Severin, Erin M. Mannen, Paul K. Edwards, C. Lowry Barnes, Jeffrey B. Stambough, Simon C. Mears

**Affiliations:** Department of Orthopaedic Surgery, University of Arkansas for Medical Sciences, Little Rock, AR, USA

**Keywords:** Prosthetic joint infection, Knee fusion, Gait analysis, Extensor mechanism failure

## Abstract

**Background:**

Extensor mechanism disruption (EMD) combined with periprosthetic joint infection (PJI) after total knee arthroplasty are life-changing complications. The literature suggests many eventually receive above-knee amputation and lose ambulatory function. An alternative is modular knee fusion (KF), but little is known about its outcomes and biomechanical function. We report early term results on a case series of patients.

**Methods:**

A retrospective review was conducted of patients who underwent 2-stage reconstruction with modular KF for combined EMD and PJI. Patient-reported outcomes at 1 year after arthrodesis and complications of surgery were recorded. Biomechanical analysis was conducted on 6 patients >1 year after surgery to measure gait speed and balance.

**Results:**

Fifteen patients received a modular KF. At the most recent follow-up visit (average 25.7 months), 12 patients had their modular KFs in place and were ambulatory while 2 had died. Six patients used a walker; 4, a cane; and 2, unassisted. Gait analysis of 6 of these patients showed variation in patterns and speed. Balance was better than historical controls treated with above-knee amputation. Average Knee Injury and Osteoarthritis Outcome Score Junior was 76 ± 11.

**Conclusion:**

Modular KF for EMD and PJI can result in successful outcomes in terms of preventing additional operations and maintaining ambulation. While speed is variable, physical testing shows this method for limb salvage may allow patients to ambulate with a gait aid although further studies are needed to evaluate midterm and long-term results.

## Introduction

Patients with combined periprosthetic joint infection (PJI) and extensor mechanism disruption (EMD) after total knee arthroplasty (TKA) are extremely challenging to manage [1]. These patients have undergone numerous previous knee surgeries, often including prior 2-stage treatment of their PJI and can have significant bone loss with soft tissue compromise. Options for management of these patients include another 2-stage attempt with extensor mechanism reconstruction (mesh vs allograft) [1], above-knee amputation (AKA) [2], or knee fusion (KF) [3]. A second 2-stage protocol with extensor mechanism reconstruction necessitates prolonged immobilization and delayed ambulation [4,5], and a recent multicenter study demonstrated a 75% rate of failure, mostly from reinfection [1]. While AKA is an option, results are also not favorable with loss of mobility, prolonged hospital stays, and high readmission rates [[6], [7], [8]]. Hungerer et al. [2] found that no patients who underwent AKA over the age of 60 years were able walk with a prosthesis.

KF is a third option. Traditional bony fusion is difficult in patients with massive bone loss as the surgeon is confronted with a significant amount of bone loss from removal of revision parts and damage to the tibial tubercle. With smaller degrees of bone loss, bony KF is an effective treatment option [3]. Modular KF uses stemmed implants and a metal segmental component to restore leg length. Such a construct can allow for immediate weight bearing and may give a better functional result than AKA [2]. Previous KF studies have examined functional scores, achievement of bony union, and patient-reported outcomes [7,[9], [10], [11], [12]]. The ability to ambulate and safely stand are likely the 2 most important functions that patients hope to regain after arthrodesis, yet there is a paucity of information regarding outcomes that quantify these tasks.

The aims of our study were to report on the early clinical, radiographic, and biomechanical outcomes, including gait speed and balance parameters, in a case series of patients who received a 2-stage segmental modular cemented fusion prosthesis for septic TKA with concurrent EMD in the setting of marked bone loss about the knee.

## Methods

After receiving institutional review board approval, we performed a single-institution retrospective review of patients who had undergone modular KF from 2016 to 2018. Patients were invited to participate in biomechanical in vivo evaluation in our gait laboratory to assess gait parameters and balance. All patients who participated in gait analysis provided informed consent.

Demographic information including age, gender, and body mass index (BMI) was collected from the electronic medical record. Clinical information including number of prior surgeries, length of intercalary piece used for modular prosthesis, pathogen isolated from surgery, ambulatory status at the most recent follow-up visit, and any further operations after ultimate arthrodesis were also documented. PJI and host status were defined using the Musculoskeletal Infection Society diagnostic criteria and staging system [13,14]. EMD was diagnosed clinically by either extensor lag >30° on preoperative evaluation or during visual inspection at the time of initial surgical debridement.

### Surgical technique

Patients selected for modular KF were indicated at the discretion of the treating surgeon based on their clinical presentation or factors found intraoperatively during initial resection. Determinants leading one toward modular fusion included the status of the local soft tissue envelope, a history of previously failed 2-stage treatment for PJI, and chronic rupture of the extensor mechanism viewed during initial debridement. Patients with irresistible hardware or severe medical comorbidities were not treated with initial debridement and typically selected AKA and are not included in this report. Patients presenting with PJI initially underwent implant removal followed by extensive debridement and placement of static antibiotic cement spacer. Static spacer constructs were variable among surgeons, but all included an intramedullary implant in combination with antibiotic-loaded polymethylmethacrylate consisting of at least 2 g of vancomycin and 2.4 g of tobramycin per 40-g pack of Cobalt bone cement (DJO Surgical, Lewisville, TX). Intramedullary implants were as follows: spine rod, 6; femoral nail, 2; humeral nail, 2; tibial nail, 1. Patients were kept non–weight-bearing in a knee immobilizer while the static spacer was in place. Systemic antibiotics were then managed by the infectious disease service for a minimum of 6 weeks. After completion of antibiotics, patients had at least a 2-week antibiotic holiday (average 8.5 weeks) and assessment of the clinic wound to aid in decision-making for second-stage surgery. The use of additional C-reactive protein (CRP) and erythrocyte sedimentation rate (ESR) testing and aspiration before reimplantation was at surgeon discretion. Fourteen modular KFs were performed with a Stryker (Kalamazoo, MI) GMRS system. The remaining patient had a revision using a Biomet (Warsaw, IN) Orthopedic Salvage System modular KF. This patient had prior treatment with modular KF by an outside surgeon for PJI and EMD and presented with a loose tibial component with proximal tibia fracture. A longer body was placed, and the tibial component was revised with a cemented stem into the distal tibia.

**Figure 1:**
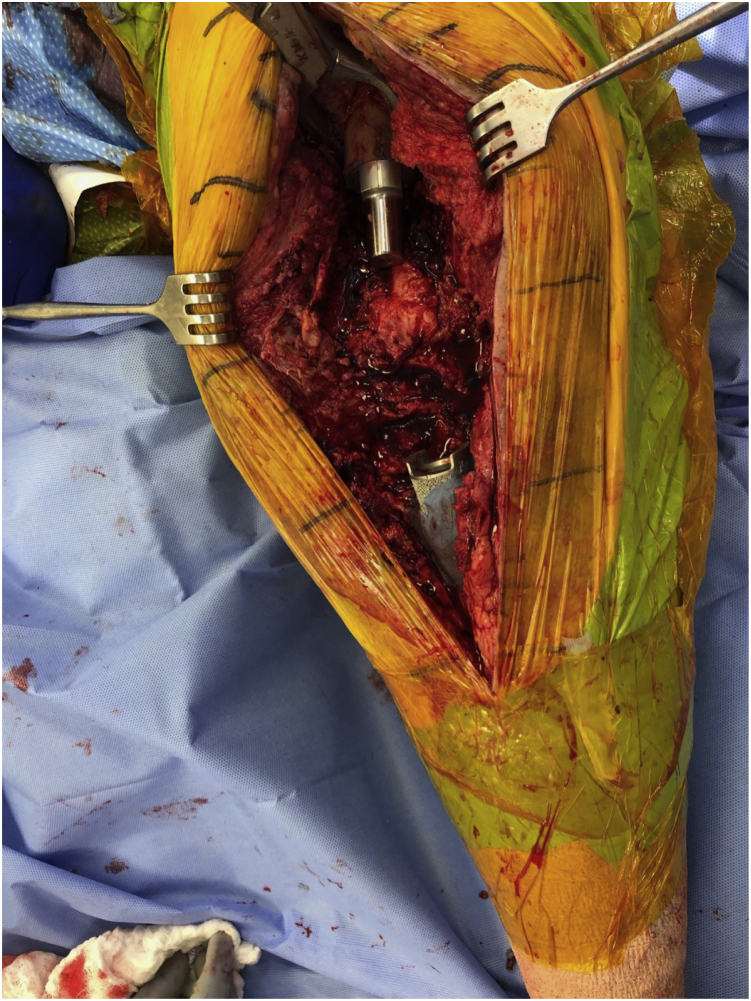
Intraoperative photograph demonstrating cementation of proximal and distal stems before placement of the intercalary bodies. Also shown is the large defect that remained after resection and antibiotic spacer placement.

**Figure 2:**
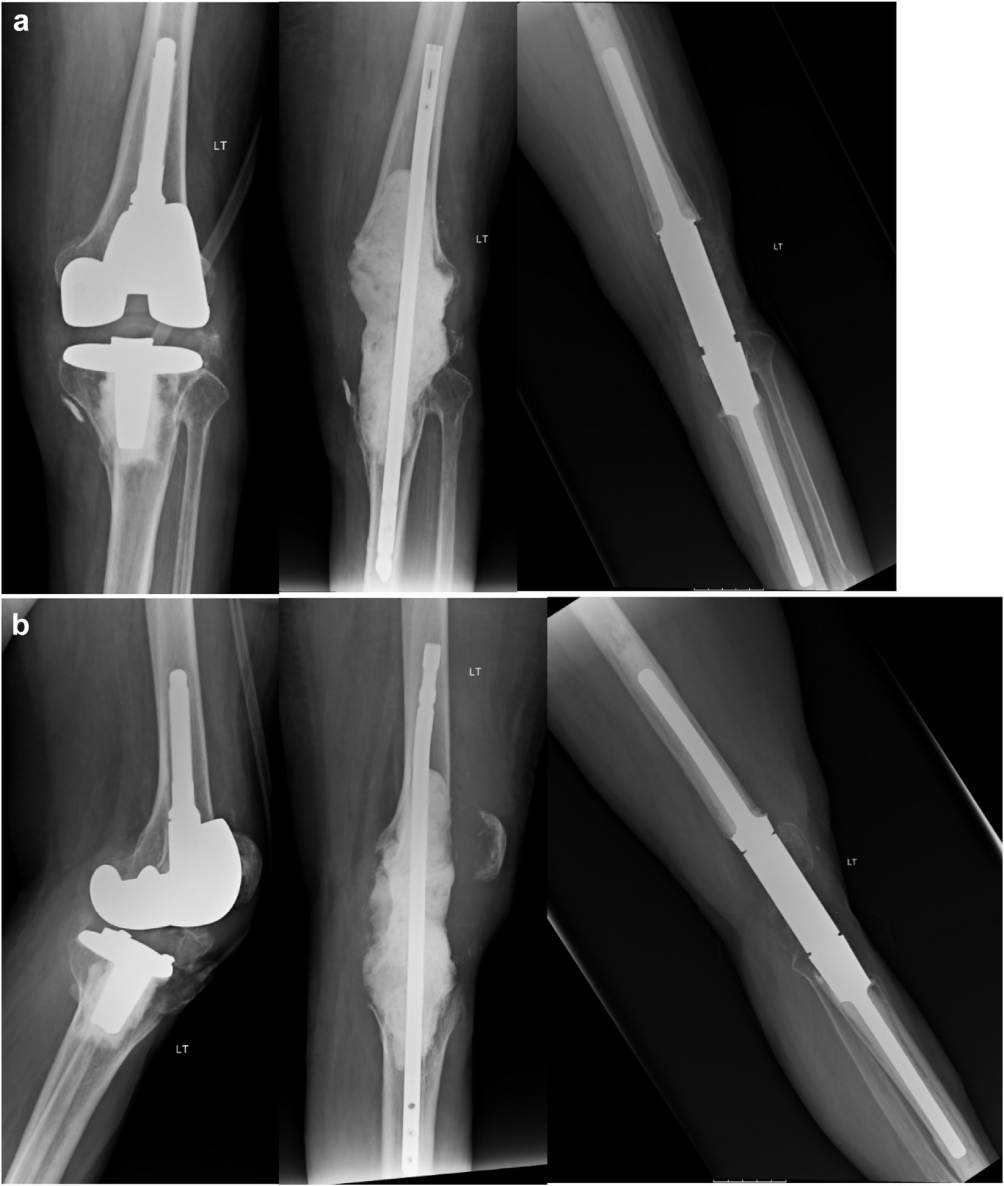
Radiographs of a representative case. Anteroposterior (a) and lateral (b) views depicting presentation, static antibiotic spacer placement, and final construct.

Bone stock lost from the distal femur and proximal tibia was replaced with intercalary bodies with the goal to recreate a leg length slightly shorter than that of the contralateral leg to allow for swing through of the operative leg with ambulation. Fully cemented stems were used in all cases with cement restrictors and pressurization except in the one revision case, which was too distal in the tibia for restrictor placement. In order to reduce the construct to appropriately mate the components, we typically implanted the male end of the Morse taper proximally in the femur and the female end in the tibia. The cement was allowed to cure before inserting the intercalary body (Fig. 1). The entire fusion system was manually reduced into place by axial pressure through the calcaneus. Further engagement of the Morse taper was achieved once the patient bore weight postoperatively. We have found that it is imperative to fully engage the Morse taper so that it does not dissociate before weight-bearing. Care was taken to not malrotate the leg during construction of the modular KF. Additional cement was placed surrounding the intercalary bodies in 5 of 12 patients in this cohort. Figure 2 shows radiographs of a representative case. Patients were allowed to fully weight-bear as tolerated immediately postoperatively without the use of an immobilizing brace. Intraoperative cultures were taken during all modular fusion stage-2 procedures and were negative. Patients were treated with 3 months of oral antibiotic after fusion surgery per the routine of Inabathula et al. [15]. Patients were followed up at regular postoperative intervals. Standard radiographs as well as standing bi-plane low-dose radiograph images were routinely obtained (EOS Imaging, Paris, France) at 1-year follow-up if patients could schedule it during their biomechanical gait analysis. Tibia and femoral cement mantle quality were graded as described by Barrack et al. [16].

Fifteen patients with EMD and PJI received modular KF (1 revision) during the study period. Demographic information for each patient is listed in Table 1. Average age at the time of modular KF was 68.5 years (range, 45-85; standard deviation [SD], 5.8). Patients had an average BMI of 38.2 kg/m^2^ (range, 20-47; SD, 5.1), and 70% were male. Before presentation to our institution, patients had undergone an average of 3.6 surgeries (range, 1-7) on the affected knee. Sizes of the modular segmental body used to span the central bone defect ranged between 30 and 270 mm, with the average being 88 mm (SD, 70.2). Of these 15, 3 were excluded from clinical analysis. Of these 3 patients, one patient was unhappy with the function of the fused knee and opted for an AKA less than a month after KF. Two other patients died in the acute postoperative period, one occurring in the immediate postoperative period from cement embolization with subsequent pulmonary embolism. The other subject died 6 weeks postoperatively because of unknown causes. This left a cohort of 12 patients for clinical evaluation with follow-up averaging 25.6 months (range, 9-48 months; SD, 13.2).

**Table 1:** Demographics.

Patient	Age	Host grade	Gender	BMI	Prior 2-stage reconstruction	Time between surgeries (wk)	Pathogen isolated at time of PJI	Outcome at last follow-up
1	72	B	F	37	No	20	*Proteus mirabalis*	Walking with walker
2	62	C	F	33	Yes	28	None	Walking with walker
3	77	A	F	38	No	8	Oxacillin-resistant *Staphylococcus aureus*	Walking with walker
4	76	B	F	38	No	8	None	Walking with cane
5	69	C	F	32	No	15	*Staphylococcus epidermidis*, *Klebsiella*	Walking with cane
6	75	B	F	37	No	9	Group B *Streptococcus*	Walking with walker
7	59	A	M	44	Yes	8	*Pseudomonas aeruginosa*	Walking unassisted
8	64	B	M	47	No	25	None	Walking with cane
9	66	A	M	42	Yes (3)	n/a	None	Walking unassisted
10	70	B	M	45	Yes (2)	11	Oxacillin-susceptible *Staphylococcus aureus*	Walking with walker
11	63	B	F	36	No	64	None	Walking with walker
12	70	B	F	33	No	13	*Staphylococcus epidermidis*	Walking with cane
13	80	B	F	20	No	8	Oxacillin-resistant *Staphylococcus aureus*	Death 6 weeks postoperatively
14	85	C	F	31	No	12	*Enterobacter cloacae*	Death postoperatively
15	45	A	M	34	No	13	Enterobacter, *Enterococcus coli*, Streptococcus	AKA

AKA, above-knee amputation; BMI, body mass index; F, female; M, male; PJI, periprosthetic joint infection.

### *In vivo* biomechanical evaluation

At the 1-year follow-up clinical visit, subjects were also offered to complete biomechanical evaluation and patient-reported outcomes report. Patients who were available and willing to travel to the laboratory during 2019 were enrolled in the study. Written informed consent was obtained before data collection. Patients completed a Knee Injury and Osteoarthritis Outcome Score Junior and Patient-Reported Outcomes Measurement Information System (PROMIS) questionnaire.

For biomechanical evaluation, 35 retroreflective markers were allocated to the participants’ trunk and lower body. Data were collected using a 10-camera motion capture system (Vicon, Oxford, UK, 100 Hz) while each participant completed 3 trials of 10-meter overground gait and one 30-second quiet standing task, where the patients stood on 2 force platforms (AMTI, Watertown, MA). The data were exported into Visual3D (C-motion) for postprocessing, and data were filtered with a cutoff frequency of 10 Hz. Spatiotemporal gait parameters were extracted from the gait trials, and center-of-pressure (COP) parameters during the quiet standing task, including mediolateral excursion, anteroposterior excursion, 95% confidence ellipse area, and path length were analyzed using a custom Matlab code (2019a; Mathworks, Natick, MA). Analysis of variables included calculation of average, standard deviation, and range.

## Results

### Clinical

After placement of modular KF, patients averaged 4.4 days in the hospital before discharge (range, 1-16; SD, 4.14). Seven patients were discharged directly home, and 5 to a post–acute care facility. No readmissions were noted to our hospital system in the first 90 days. At the latest follow-up visit, 6 patients were noted to use a walker, 4 patients a cane, and 2 patients were walking unassisted.

Three patients had reoperations. One patient required a revision of her femoral component because of loosening at 4 years after initial arthrodesis. Intraoperative cultures had an initial positive Gram stain for gram-positive cocci. However, all 3 final cultures were found to be negative. She was discharged home with intravenous antibiotics for 6 weeks. She has had no further revisions now 8 months postoperatively. Another subject developed knee pain and inability to walk 14 months after his revision modular KF. Radiographs showed a fracture of the implant at a transition point in the intercalary piece. He was treated with replacement of a new intercalary segment. This was the only patient in our series with a revision modular KF using the Biomet Orthopedic Salvage System. Contributing factors to the implant breakage included the very long intercalary body (110 mm), his relatively high activity level, and his BMI of 43 kg/m^2^. The third patient was a 45-year-old male who was unhappy with KF and underwent AKA less than 1 month after surgery. At the most recent follow-up visit, none of the subjects in our series demonstrated clinical signs of persistent or recurrent infection, and no one remained on suppressive antibiotics.

**Figure 3:**
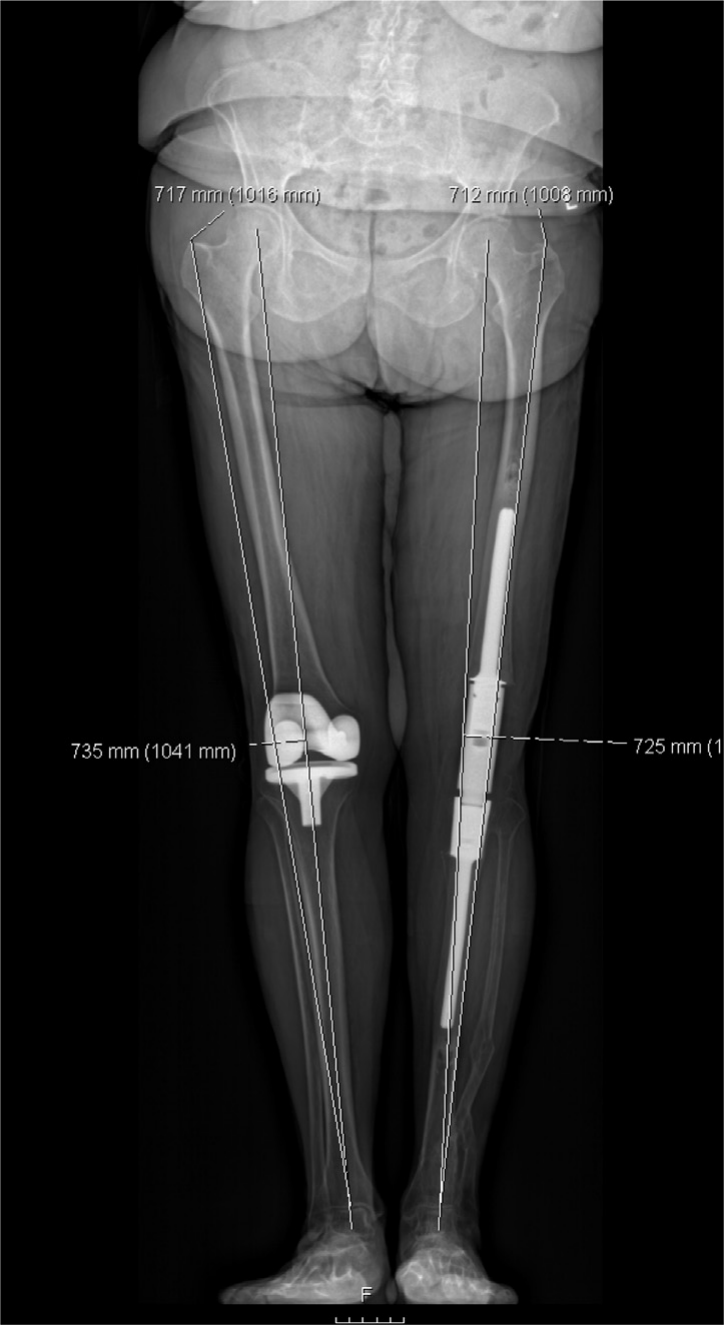
Coronal EOS imaging of same patient from the representative case in Figure 1 at 1 y postoperatively.

Radiographs of the modular knee constructs were reviewed at their most recent clinical follow-up visit (average, 16 months; SD, 12). Patient1 had a revision for femoral loosening. Of the remaining prostheses, the femoral cement mantle grades were as follows: A = 4, B = 7, C = 0, D = 1. Tibial stems were rated as A = 5, B = 2, C = 4, D = 1. The 2 D grades were secondary to cement mantles not extending past the distal aspect of the stem. None of these stems were deemed radiographically loose. In reviewing the available EOS scans (7/12), the operative leg was 8.4 millimeters (range, −44 to 13 mm) shorter than the contralateral limb. This measurement was determined from the tip of the greater trochanter proximally to the tibial plafond distally (Fig. 3).

### Biomechanical

Six of the 12 subjects (male = 2, female = 4, age = 69 ± 5 [64-77] years, BMI = 39.5 ± 7.8 [30.0-51.2] kg/m^2^), 12 ± 1 months after the operation (L = 3, R = 3), opted to participate in the in vivo biomechanics portion of the study. Patient-recorded outcomes scores were measured in the 6 patients who participated in the biomechanical portion of the study. The average Knee Injury and Osteoarthritis Outcome Score Junior score was 76 (SD, 11; range, 66.1-91.4), and PROMIS physical function was 37 (SD, 11.9; range, 22.9-56.9). PROMIS scores for anxiety, depression, fatigue, sleep, social isolation, and pain were all less than one standard deviation from the adult average (anxiety, 49.6; depression, 46.2; fatigue, 53.7; sleep, 53.8; social isolation, 41.2; pain, 57.9). All subjects were able to complete overground walking and 30 seconds of quiet standing. There was great variation of spatiotemporal gait parameters including gait speed, cadence, stride distance, and double stance time. Less variation was seen in COP parameters from the 30-second quiet standing task (Table 2).

**Table 2:** Biomechanical gait analysis.

Patient	KOOS JR	PROMIS physical function	Speed (m/s)	Stride width (cm)	Cadence (steps/min)	Double support (%)	Leg length discrepancy (mm)
5	91.4	56.9	0.82	13.81	105.81	36	−5
6	75	22.9	0.26	29.58	71.74	60	13
8	66.1	32.1	0.92	17.70	92.68	34	12
9	72.7	39.1	0.25	20.67	65.05	54	−44
11	58	29.1	0.25	22.60	56.84	55	2
12	69.1	41.8	0.43	19.20	69.40	46	−33
Average	76 ± 11 (58-91.4)	37 ± 11.9 (22.9-56.9)	0.5 ± 0.3 (0.2-0.9)	20.6 ± 5.3 (13.8-29.6)	76.9 ± 18.5 (56.8-105.8)	47.6 ± 0.1 (34-59.8)	−8 ± 22 (−44 to 13)

KOOS-JR, Knee Injury and Osteoarthritis Outcome Score Junior; PROMIS, Patient-Reported Outcomes Measurement Information System.

## Discussion

We have demonstrated a preserved ambulatory ability in patients with a devastating problem of combined PJI and EMD through the use of modular KF constructs with large intercalary segments. Previous techniques for limb salvage using revision knee replacement and extensor mechanism reconstruction have mixed success reports of very high failure rates [1].

Despite the relative success in our specific cohort, this surgical technique is not without risk. One patient died of apparent cement embolism immediately after surgery while another died of unknown reasons 6 weeks after surgery. It should be noted that a recent review of Medicare patients showed a 28% decrease in mortality if patients being treated for TKA PJI underwent some sort of salvage procedure vs undergoing amputation [8]. One of the patients was also unhappy with the straight knee and underwent AKA, and he happened to be the youngest patient in the study (45 years old). Older age at amputation has been shown to significantly lower functional outcomes and quality of life [7]. Furthermore, the use of a long modular body puts significant biomechanical stress on the device itself as well as the cement stem interface, so this is not an attractive option for younger patients with longer life expectancies. One subject who had a modular fusion with a system with a smaller trunnion had his trunnion break. This patient was also very active, had a high BMI, and had a very long central modular segment. There is a concern that using this construct can lead to aseptic loosening, so longer term follow-up is certainly needed. Another option for fixation could be the use of uncemented stems, especially if tapered fluted stems were to be developed for similar fusion constructions, as previously described in custom situations [17].

Functionally, older patients do not do as well after an AKA vs arthrodesis [2]. Limb salvage with arthrodesis does have higher reported rates of recurrent infection than amputation but with greater functional benefits. In comparing attempted 2-stage revision vs arthrodesis, Gathen et al. [18] noted lower revision rates in those treated with arthrodesis. Their indication for proceeding with arthrodesis was poor local soft tissues and deficient extensor mechanism, similar to our criteria. Elderly patients who have several comorbidities had lower incidence of prosthesis use after AKA and, thereby, lower functional status. We feel the greatest benefit of limb salvage with arthrodesis, regardless of the amount of bony deficits, is a somewhat preserved functional status and lower revision rate. The highest functioning subject in our series reported a PROMIS physical function score of 56 and demonstrated gait parameters similar to those of healthy older adults. However, the average physical function achieved in our group was over one standard deviation below that of the average adult (PROMIS PF = 37; range, 22.9-56.9), signifying realistic limits for actual functional levels. PROMIS scores for other outcome measures were slightly lower than average.

The use of a modular KF allows for maintenance of limb length compared with a traditional bony fusion, which is particularly important in cases with extensive tibial and femoral bone loss. Bony fusions using external fixators, multiple plates and screws, or intramedullary rods have been successful in patients with good bone stock [3,19]. Newer devices to the market have a metal spacer to replace periarticular bone loss but are anchored to the diaphyseal bone via interlocking screws. Several modular implants have been described as well, but these implants do not have options for intercalary segments to span bony deficits greater than 50 mm (Endo-Model Knee Fusion Nail SK Modular System; Waldemar-Link, Hamburg, Germany, and OsteoBridge, Merete Limb Salvage Systems; Merete Technologies Inc., Oakbrook Terrace, IL). Patients in our cohort had an average intercalary piece length of 87 mm, which is longer than the segments reported by Iacono et al. [10] and Putman et al. [20]. Others have reported high success rates using modular implants without bone-to-bone contact, as Friedrich et al. [21] showed an 86.5% survivorship with midterm follow-up using the 2-stage fusion reconstruction technique. Namdari et al. [22] reported successful midterm follow-up of a single case using a similar large modular construct similar to ours to bridge significant bone loss with a 160-mm intercalary piece for modular arthrodesis.Certainly, longer modular construct gives added stress to the stem bone interface, the trunnion, and the implant themselves and may be at risk for fracture or breakage in the future. Longer term follow-up will be necessary to determine this risk.

The biomechanical evaluation of patients in our study is unique in that it provides added understanding of quantitative functionality of these fusion recipients during standing and walking. While our biomechanical results for the 6 subjects showed significant variability in both the gait and balance parameters, this is not considerably different compared with age-matched, healthy older adults [23].When comparing the overall function of our participants to other salvage operations, all participants who received modular KF could ambulate and demonstrate superior biomechanical outcomes compared with reports of those receiving AKA (Table 3). This corroborates other studies that have compared the outcomes of AKA and arthrodesis [2,6,7]. All subjects examined in the in vivo biomechanics portion of the study were able to complete overground walking, which is encouraging considering AKA surgery would have most likely left these patients wheelchair bound [19,23,24]. The lower gait speed and cadence coupled with greater stride width and double support for the arthrodesis participants compared with able-bodied adults [25,26] was expected because of the inability to bend the surgical knee joint.

**Table 3:** Comparison of balance parameters.

Balance parameter	Arthrodesis	TF amputation [27]	Healthy
COP AP (cm)	1.7 ± 0.7	3.3 ± 1.5	3.1
COP ML (cm)	2.8 ± 0.6	2.6 ± 1.4	3.0
95% CI ellipse area (cm^2^)	2.3 ± 1.3	–	–
COP length (cm)	58.5 ± 15.3	70.2^a^	81

AP, anteroposterior; ML, mediolateral.

Center-of-pressure (COP) parameters for patients with unilateral knee arthrodesis, patients with transfemoral (TF) amputation, and healthy older adults.

a Adjusted to 30s for comparison.

The COP analysis during standing showed that patients who had undergone the salvage arthrodesis surgery had postural sway parameters comparable to those of healthy, age-matched controls [28] and less anteroposterior and mediolateral excursion than patients with transfemoral amputation [27]. This suggests that modular arthrodesis surgery enables patients to maintain proprioception, which affords benefits to balance during standing and gait. In all, the broad range of 1-year patient-reported outcome scores, postural sway measures, and gait parameters are indicative of the variation in preexisting functional abilities of this diverse patient population.

Our study is not without limitations. Given the rarity of this clinical scenario, our patient numbers are small, and broad generalizations are difficult to make as there is no direct comparison group. It would likely take a multicenter cohort study to gather a large-enough sample size to potentially randomize patients to various knee salvage treatments to provide adequately powered comparison groups. Second, because of our status as a tertiary referral, we did not have presurgery patient-reported outcomes because most patients were transferred to our hospital to assume a higher level of care for the stage-one explanation. Thus, our ability to precisely determine the relative amount of patient improvement is hindered. Third, we only report 1-year radiographic and clinical outcomes in this unique patient group, so it is possible for cement mantle loosening or modular junction failure to occur in the future. This is particularly worrisome in our cohort because all patients who we have followed up were obese (BMI, >30 kg/m^2^), with the average BMI approaching morbid obesity levels (BMI = 40 kg/m^2^), and those who achieved a higher level of function level will theoretically place greater stresses across the modular and cement interfaces, thus predisposing to loosening. Long-term surveillance is essential for these patients. Fourth, patients with modular KF are at risk for return of prosthetic joint infection. AKA would provide a more likely chance of infection eradication. Finally, it should be noted that the described surgical arthrodesis is not without risk, as 2 subjects experienced early mortality, one of which was from presumed bone cement implantation syndrome intraoperatively. This highlights the importance of providing patients proper informed consent and discussing the potential risks. Subjects should understand that arthrodesis surgery is a last-ditch effort for limb salvage in those adamant to avoid amputation and keep their limb knowing their knee will never flex again.

## Conclusion

Our short-term outcomes of large-segment modular KF for management of concurrent PJI and EMD demonstrate that patients uniformly maintain some ability to ambulate with variable gait and balance parameters. We believe that this surgical technique affords patients a functional limb on which to stand even in the setting of significant bone loss about the knee.

## Conflict of interests

C. L. Barnes received royalties from DJO, Medtronic, and Zimmer; is a paid consultant to Health Trust, Medtronic, and Responsive Risk Solutions; has stock or stock options from Responsive Risk Solutions; received research support from ConforMIS; received other financial or material support from Corin-Non-PI; is in the Medical/Orthopaedic publications editorial/governing board of JOA and JSOA; and is a board member in AAHKS, HKA Foundation, MAOA, and SOA. E. M. Mannen received research support from Medtronic. J. B. Stambough is in the Medical/Orthopaedic publications editorial/governing board of Journal of Arthroplasty; and is a board member in American Association of Hip & Knee Surgeons (AAHKS), Education Committee, and AJRR, Steering Committee. P. K. Edwards received royalties from DJO and is a paid consultant for DJO. S. C. Mears is in the Medical/Orthopaedic publications editorial/governing board of Geriatric Orthopaedic Surgery and Rehabilitation and is a board member in International Geriatric Fracture Society.

## Appendix A Supplementary data

Conflict of Interest Statement for Severin

Conflict of Interest Statement for Stambough

Conflict of Interest Statement for Edwards

Conflict of Interest Statement for Mears

Conflict of Interest Statement for Mayes

Conflict of Interest Statement for Barnes

Conflict of Interest Statement for Mannen
