# Supplementary material for: Management of Periprosthetic Joint Infection and Extensor Mechanism Disruption With Modular Knee Fusion: Clinical and Biomechanical Outcomes
Source: Arthroplast Today. 2021 Feb 26;8:46–52. doi: 10.1016/j.artd.2020.12.008 (PMC7921710; doi:10.1016/j.artd.2020.12.008)
Supplement: Conflict of Interest Statement for Barnes [file mmc6.docx]

# CONFLICT OF INTEREST STATEMENT

***American Association of Hip and Knee Surgeons***

(Adopted from the American Academy of Orthopaedic Surgeons disclosure statement)

The following form**must be filled out completely and submitted by each author (example, 6 authors, 6 forms).**

**All items require a response. If there is no relevant disclosure for a given item, enter "*None*.”**

**Manuscript Title:**

1. Royalties from a company or supplier (The following conflicts were disclosed)

DJO, Medtronic, Zimmer

2. Speakers bureau/paid presentations for a company or supplier (The following conflicts were disclosed)

None

3A. Paid employee for a company or supplier (The following conflicts were disclosed)

None

3B. Paid consultant for a company or supplier (The following conflicts were disclosed)

Health Trust, Medtronic, Responsive Risk Solutions

3C. Unpaid consultants for a company or supplier (The following conflicts were disclosed)

None

4. Stock or stock options in a company or supplier (The following conflicts were disclosed)

Responsive Risk Solutions

5. Research support from a company or supplier as a Principal Investigator (The following conflicts were disclosed)

ConforMIS

6. Other financial or material support from a company or supplier (The following conflicts were disclosed)

Corin-Non-PI

7. Royalties, financial or material support from publishers (The following conflicts were disclosed)

None

8. Medical/Orthopaedic publications editorial/governing board (The following conflicts were disclosed)

JOA, JSOA

9. Board member/committee appointments for a society (The following conflicts were disclosed)

AAHKS, HKA Foundation, MAOA, SOA

**Each author must sign AND print or type his/her name, date and submit a separate form**

In addition, one BLINDED Conflict of Interest form (no author names used) should be submitted per manuscript with all author disclosures.

C. Lowry Barnes, MD C. Lowry Barnes, MD September 20,2020

Author Name (Print or Type) Author Signature Date
